# Implementation of Telemedicine for Patients Referred to Emergency Medical Services

**DOI:** 10.3390/epidemiologia6030036

**Published:** 2025-07-11

**Authors:** Francesca Cortellaro, Lucia Taurino, Marzia Delorenzo, Paolo Pausilli, Valeria Ilardo, Andrea Duca, Giuseppe Stirparo, Giorgio Costantino, Filippo Galbiati, Ernesto Contro, Guido Bertolini, Lorenzo Fenech, Giuseppe Maria Sechi

**Affiliations:** 1 Agenzia Regionale Emergenza Urgenza Headquarters (AREU HQ), 20124 Milano, Italyl.taurino@areu.lombardia.it (L.T.); m.delorenzo@areu.lombardia.it (M.D.); g.stirparo@areu.lombardia.it (G.S.);; 2 Pronto Soccorso e Medicina D’Urgenza, Fondazione IRCCS Cà Granda Ospedale Maggiore Policlinico, 20122 Milan, Italy; giorgio.costantino@policlinico.mi.it; 3 Dipartimento di Scienze Cliniche e di Comunità, Università degli Studi di Milano, 20122 Milan, Italy; 4 Pronto Soccorso e Medicina d’Urgenza, ASST Grande Ospedale Metropolitano Niguarda, 20162 Milan, Italy; filippo.galbiati@ospedaleniguarda.it; 5 Pronto Soccorso e Medicina D’Urgenza, Fondazione IRCCS San Gerardo dei Tintori, 20900 Monza, Italy; 6 Laboratorio di Epidemiologia Clinica, Istituto di Ricerche Farmacologiche Mario Negri IRCCS, 24126 Bergamo, Italy; guido.bertolini@marionegri.it; 7 Healthcare Management Government Course, Università Bocconi, 20122 Milan, Italy

**Keywords:** telemedicine, emergency medical system, crowding, input control, pre-hospital emergency care, emergency department

## Abstract

**Background:** he surge in the use of Pre-hospital Emergency Medical Systems (EMS) and Emergency Departments (ED) has become a pressing issue worldwide after the COVID-19 pandemic. To address this challenge, we developed an experimental and innovative care pathway supported by telemedicine. The aim of this study is to describe the activity of the Integrated Medical Center (CMI): a new telemedicine-based care model for patients referring to the Emergency Medical System. **Methods:** A prospective observational study was conducted from January 2022 to December 2022. The CMI was established to manage patients referring to the Emergency Medical System. **Results:** From January to December 2022, a total of 8680 calls were managed by CMI, with an average of 24 calls per day. 6243 patients (71.9%) were managed without ED access of whom 4884 patients (78.2%) were managed through telemedicine evaluation only, and 1359 (21.8%) with telemedicine evaluation and dispatch of the Home Rapid Response Team (HRRT). The population treated by the HRRT exhibited a higher age. The mean satisfaction score was 9.1/10. **Conclusions:** Telemedicine evaluation allowed for remote assessments, treatment prescriptions, and teleconsultation for HRRT and was associated with high patient satisfaction. This model could be useful in future pandemics for managing patients with non-urgent illnesses at home, preventing hospital admissions for potentially infectious patients, and thereby reducing in-hospital transmission.

## 1. Introduction

During the last 10 years, the utilization of Pre-hospital Emergency Medical Systems (EMS) and Emergency Departments (ED) by citizens has witnessed a significant surge [1,2]. This upsurge can be attributed to various factors, including an increase in the age and vulnerability of the population [3,4], as well as to a rise in non-urgent assistance requests [2].

Over the past 10 years, the Lombardy region has experienced a consistent annual increase of 3% in emergency assistance requests prior to the pandemic. Remarkably, in 2022, the EMS was responsible for 22% of all Emergency Department visits, with a total of 620,000 missions. Notably, 34% of all the missions dispatched by the emergency medical service (118) are identified by the first call receiver as low acuity codes; this percentage increases to 56.8% if we consider the evaluation made at triage at the arrival in the ED [5].

The high number of patients calling with acute non-emergent conditions significantly strains the EMS. It contributes to overcrowding in emergency departments, which has been further exacerbated since the onset of the COVID-19 pandemic by prolonged length of stay (LOS) and severe shortage of healthcare personnel in Emergency Departments [6].

To mitigate overcrowding and reduce the risk of COVID-19 transmission, it has become imperative to explore clinical pathways to manage non-emergency acute cases outside the ED. In line with the guidelines outlined by the Italian Ministry of Health (DM 77, May 2022) [7,8], novel care models advocating telemedicine and home care have emerged. These models seem particularly promising for vulnerable patients, as home treatment prevents complications associated with hospitalization and enhances their quality of life and satisfaction, along with that of their caregivers [9].

Telemedicine in the last decades has garnered substantial experience internationally. Notably, within the pre-hospital setting, telemedicine has become a well-established tool for managing time-dependent conditions, facilitating seamless collaboration between hub and spoke hospitals, and enabling professional consultation [10,11,12]. Its effectiveness has been demonstrated in optimizing treatment timelines for stroke and ST-elevation myocardial infarction (STEMI) patients, reducing centralization and improving treatment initiation [13,14,15,16,17]. Furthermore, telemedicine ensures the appropriateness of secondary transfers to hub facilities [18]. Experiences exist where paramedics use telehealth and teleconsultation to enhance pre-hospital care, receiving remote support from medical professionals [19]. Teleconsultation is crucial in remote areas as a supportive mechanism for diagnostic and therapeutic processes through seamless data and image exchange [20]. However, most studies focus on supporting paramedics on scene rather than on the complete remote management of the patient through telemedicine. The development of an alternative pathway to on-site assessment for patients calling the emergency medical service would allow for resource savings and reduce discomfort for patients [21].

The Lombardy region’s emergency response system consists of four Regional Emergency and Urgency Operation Centers (SOREU) strategically located across the territory (Metropolitan SOREU, Lakes SOREU, Alps SOREU, Plain SOREU). The SOREU is contacted through the emergency hotline number 112. Initial triage and call management are performed by trained non-medical personnel (first responders) who assign a severity code (white, green, yellow, red) to each call using a predefined filtering system. This severity code determines the appropriate level of resources and priority for dispatch. After the arrival of paramedics on the scene, a console comprised of nurses and physicians directs the ambulance to the most suitable hospital resource available.

The Lombardy regional healthcare system is part of the Italian National Health Service, which is a state-funded universal healthcare system financed through taxation. Patients who call the emergency number (112) and are subsequently assessed and treated do not have to pay any out-of-pocket costs.

This study aims to describe the activity of the Integrated Medical Center (CMI): an innovative care pathway for patients calling the EMS with non-emergent conditions employing telemedicine assessments with eventual activation of local resources in selected cases.

## 2. Materials and Methods

The study was designed as a prospective, observational, single-center study.

The CMI, which was established in Lombardy on 5 January 2022, included in this study all patients referred from its inception through 31 December 2022. Patient enrollment began with the Metropolitan SOREU and was subsequently extended to the Alpine and Lakes SOREU, resulting in a cumulative catchment area serving a population of approximately 8.5 million people.

The CMI was staffed by a senior emergency physician and a technician from 8:00 a.m. to 6:00 p.m., seven days a week. During off-hours the calls were managed by the SOREU as usual, with the dispatch of an ambulance. All personnel involved in the project underwent comprehensive training tailored to their respective roles. The structure hosting the CMI was set within the Metropolitan SOREU, in a specific area designed and acoustically optimized to ensure optimal performance.

In order to develop the activity of CMI, primary clinical complaints of non-urgent patients seeking medical assistance were assessed through an in-depth analysis of calls to the emergency hotline (112) and the diagnosis of low acuity codes discharged from the emergency departments. Based on this demand map, inclusion and exclusion criteria for transferring the calls from SOREU to CMI were defined. Subsequently the activity of CMI was focused on adult patients with white/green severity codes identified by the SOREU, based on predefined inclusion and exclusion criteria (Table 1 and Table 2).

Based on the selected panel of clinical complaints, a team of experienced emergency physicians developed clinical decision pathways to guide the evaluation and management of the most common reasons for these calls. These clinical algorithms were integrated into the telemedicine platform, devised by the Regional Emergency and Urgency Operation Centers (AREU) for this experimental project. Each clinical pathway comprises an initial core section, adhering to the universally recognized ABCDE principles, and a subsequent specific section tailored to the symptoms requiring investigation and the early identification of pertinent red flags. An example of the COVID-19 flow-chart embedded in the CMI software(1.0) is provided in Supplementary Materials.

Once the first responder identifies the 112 call as suitable to be transferred to CMI, the patient is referred to the CMI; the physician working in CMI calls the patient back and establishes the need for televisit. The decision whether to use only a phone call or to include a video call as well was left to the physician’s discretion. Through a messaging system integrated into the platform, the CMI physician sends the medical report to the patient and his primary care physician, along with any necessary diagnostic and therapeutic prescriptions. Additionally, the patient can be connected to local services, such as COVID hotspots or outpatient pain clinics for lower back pain.

When additional patient assessment in the emergency department (ED) is considered necessary, an emergency response vehicle may be dispatched, or the patient may be advised to self-refer to the ED. If diagnostic investigations or treatments are required—particularly for frail patients—but there is no urgent need for hospital admission, the “Home Rapid Response Team” (HRRT) can be activated and sent directly to the patient’s location. The HRRT consists of a medical doctor with at least two years of training in Emergency Medicine and is equipped with point-of-care diagnostic tools, including ECG, blood gas analysis, ultrasound, and basic laboratory tests (electrolytes, lactate, renal function, and hemoglobin). During interventions, the HRRT can consult remotely with the CMI physician for additional support. Continuous quality monitoring of the service is conducted through periodic case review meetings, involving calls and analysis of CMI activity.

The Ethics Committee of Fondazione IRCCS Ca’ Granda Ospedale Maggiore Policlinico of Milan n° 656_2022 approved the study.

Data were extracted from the CMI application database, while demographic and clinical information for the enrolled population were obtained from AREU’s dedicated platform. Among the non-demographic variables, clinical complaints and outcomes—including ambulance dispatch, self-referral to the emergency department, telemedicine visits, and HRRT dispatch—were analyzed. The final disposition following the evaluation (remaining at home, HRRT activation, or referral to the emergency department) was also recorded. Regarding the population evaluated by the Rapid Response Home Team, data were collected about tests performed during the home visit, and outcomes (ambulance dispatch, self-referral to the emergency department, telemedicine visit).

Each patient consented to telemedicine evaluation and a phone follow-up on recorded lines (as per the Ethics Committee). All patients were called back for follow-up after ten days from CMI evaluation, except the ones sent to the emergency department; patients were asked about their compliance to prescriptions, satisfaction with the service and whether they received further care from their general practitioner after CMI management.

Statistical analysis was carried out by STATA software (18). Continuous variables are expressed as mean and standard deviation. Student T-test was use to compare continuous variables with normal distribution. All statistical tests were 2-tailed, and statistical significance was defined as *p*  <  0.05.

## 3. Results

From 5 January 2022, to 31 December 2022, the CMI managed a total of 8680 calls, with an average of 24 calls per day (SD: 10.0). The number of events managed by the SOREU involved in the project in the same time frame was 550,324. The mean age of patients managed by CMI was 63.4 years (SD: 22.5); 55% were over 65, and 32% were over 80. Out of this population 53.5% of patients were female and 46.5% were male. The most prevalent complaint was COVID-19, followed by fever and back pain (Table 3).

**Figure 1:**
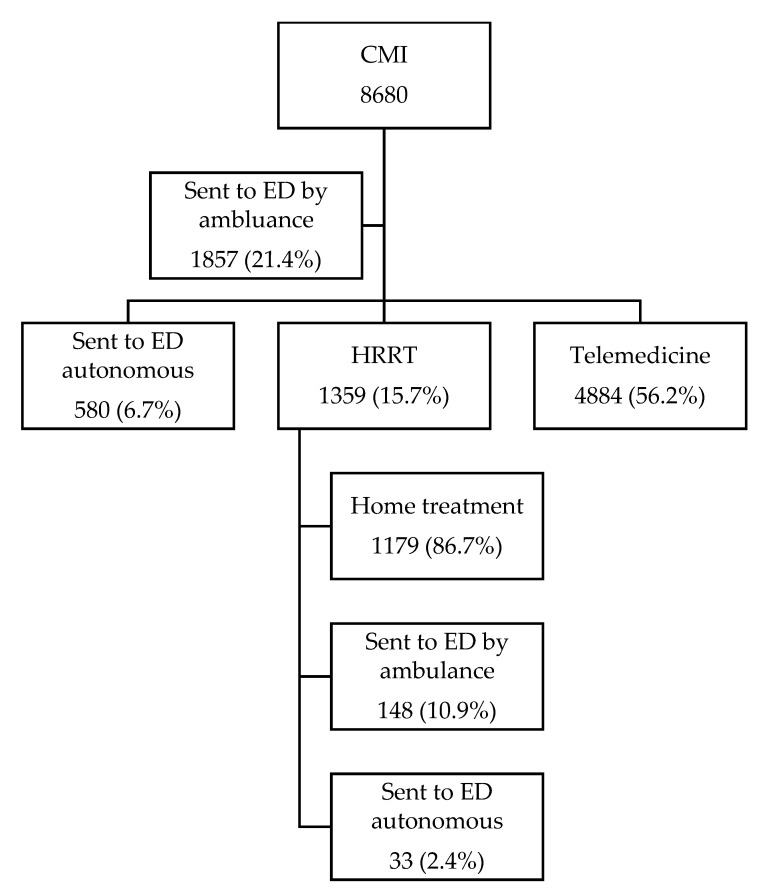
Outcomes of patients managed by CMI.

After the medical assessment in CMI, 1857 (21.4%) patients were sent to the ED by ambulance and 580 (6.7%) autonomously (Figure 1). Among the remaining 6243 patients (71.9%), 4884 patients (78.2%) were managed only through telemedicine evaluation, with the prescription of medication in 30% of cases and in the remaining 1359 patients (21.8%) telemedicine evaluation was followed by dispatch of the HRRT (Figure 1).

### 3.1. HRRT Population

From 5 January 2022, to 31 December 2022, HRRT evaluated at home 1359 patients. The mean age of patients evaluated by the HRRT was 69.8 years (SD: 21.2), compared to 63.3 years (SD: 22) of those treated in CMI through telemedicine alone (*p* < 0.001); 66% of the patients were over 65, 44% were over 80. Out of this population 54.7% of patients were female and 45.3 were male.

Similarly to the general population in CMI, COVID-19 and fever were the most prevalent complaints (Table 4). The diagnostic procedures performed on these patients were as follows: point-of-care ultrasound (POCUS) in 54% of patients, blood tests and arterial blood gas analysis (BGA) in 18% of patients, rapid antigen testing for SARS-CoV-2 in 44% of patients, and parenteral medications administered in 60% of patients.

Following the HRRT’s home evaluation, 148 (10.9%) patients were sent to ED by ambulance and 33 (2.4%) were advice self-referral. The remaining 1179 patients (86.7%) were treated at home (Figure 1).

### 3.2. Follow-Up Activities

Only 2148 patients (31.5%) of patients not sent to the emergency department by ambulance answered the follow-up call.

During the telephone follow-up, complete compliance to the instructions provided by CMI was reported in 93.5% of cases. Additionally, 74.8% of patients stated they had contacted their general practitioner. The mean satisfaction rate was 9 (SD: 1.7) on a scale of 0 to 10.

## 4. Discussion

During its activity, the CMI avoided the evaluation in the ED of 6243 patients, with one-third of the patients managed falling into the age group over 80. This group generally includes frail patients at high risk of complications related to hospitalization, for whom community-based management represents a valid alternative to hospital admission, reducing the risks associated with hospitalization (e.g., nosocomial infections, falls, delirium, bedridden syndrome, pressure ulcers) [22]. The most frequently managed clinical issue in CMI was SARS-CoV-2 infection, particularly during the pandemic waves in January-February and July, followed by fever and lower back pain. In this study, telemedicine was employed throughout the patient care process, including teleconsultations in CMI, HRRT teleconsultations with the CMI physician, and teleconsultations with infectious disease specialists for targeted therapies.

There are currently few experiences in the literature regarding home treatment for patients who initially call the Emergency Medical System. One significant experience was initiated in London with the creation of the Physician Response Unit (PRU) [23]. The PRU team, activated by the London Ambulance Service Emergency Operations Centre (LAS EOC), consists of a senior emergency medicine doctor working alongside an emergency ambulance crew clinician. The PRU is activated for calls related to medical and trauma emergencies, as well as for home treatment of non-urgent issues. In 12 months, this team managed 1924 patients, treating 1289 of them at home (67% of the total). The PRU promotes the Community Emergency Medicine model, aiming to transport to the hospital only those who are believed to require hospitalization. Another noteworthy experience is the Alternative Pre-Hospital Pathway (APP) Team, created in Ireland for managing low-acuity emergency calls [24]. This team comprises a Specialist Registrar (SpR) in Emergency Medicine and a National Ambulance Service (NAS) emergency medical technician (EMT) in a NAS response vehicle. Activated by the dispatch center, the team managed 2001 minor cases in 12 months, with a non-conveyance rate of 67.8% [24]. A study conducted by the Dublin ED proposed sending a frailty response team to nursing homes in response to emergency calls, thereby avoiding the transfer of 549 out of 592 patients to the ED in 105 days [25].

While the CMI experience aligns with the intentions of previous studies to improve the appropriateness of patients’ ED visits, it is the study with the highest number of recruited patients and, particularly, it is the first study to our knowledge, proposing a patient management model initially based on telemedicine. The CMI provided care for over half of the patients without utilizing additional resources, allowing remote treatment and prescriptions.

Various models are described in the literature for managing patients in the community, specifically those who do not require assessment in hospital. This opportunity is possible according to the medical professionals employed in the Emergency Urgency system, especially according to the training and skills of the personnel [26,27,28], in fact we decided to use doctors with experience in emergency emergencies. In addition, an advantageous element compared to other experiences is the presence of a doctor at the dispatch center who manages the patient already in the call phase, saving the ambulance dispatch to the patient’s home, this is advantageous compared to other studies where the assessment was carried out by paramedics at the patient’s home [29,30,31,32].

The emergency network plays a crucial role in healthcare systems by providing real-time data on the health status of the population. Information collected from Emergency Departments (ED) has been integrated into surveillance systems and serves as a valuable indicator of the general health of the community. This data guides decision-makers in planning, managing resources, and addressing emerging health needs. The introduction of a central unit, such as the Medical Information Center (MIC), can be seen as an enhancement to the Emergency Medical Services (EMS) system. It ensures more efficient resource allocation, improving the overall responsiveness and effectiveness of healthcare delivery [33,34]. The emergency system is strongly influenced by seasonality, and respiratory infections are correlated with an increase in calls and missions, so the development of power plants is a useful element in reducing the commitment of these systems [34].

In our study, the category of patients requiring the HRRT evaluation had a higher mean age and required further diagnostic investigation in half of the cases and medication administration in 60% of cases.

The preliminary telemedicine assessment by the CMI physician allowed proper patient stratification, referring only a fraction of the patients to the HRRT, particularly prioritizing fragile and complex patients.

HRRT is dedicated to low acuity emergency calls, and compared to PRU, which also handles major emergency calls, requires less expenditure of resources in terms of training, staffing and equipment, allowing for easier recruitment, especially at this stage of dramatic shortage of emergency physicians.

Most patients managed by the CMI presented with symptoms related to respiratory infections. This finding is very relevant, even if not specifically evaluated in this study, since the remote management of these patients could also be a useful tool for reducing the spread of these pathogens in the hospital setting, while protecting other patients who might be admitted to hospital. in fact, as is well known, during COVID there was a great spread of airborne pathogens even in the hospital setting, and early management could have reduced the moments of contagion in the emergency room.

The high degree of patient satisfaction (9/10, SD 1.7), although estimated from a low response rate, shows that the use of telemedicine was considered by patients to be a valid and satisfactory tool, even in the face of an initial request for urgent hospitalization.

Our study shows the feasibility of a new organizational model in the home treatment of non-emergent acute problems by telemedicine, allowing optimization and reallocation of the available resources.

The most significant limitation of the present observational study is due to the low response rate of follow-up calls, not allowing to evaluate properly the safety of this new strategy. Moreover, the setting in which this study was conducted is unique and closely linked to the distinctive organization of the Lombardy emergency medical system, which involves the use of non-medical personnel as first receivers in the SOREU and paramedics whose training is considerably more limited than in other countries. Additionally, the training of the HRRT includes skills typically associated with the discipline of emergency medicine, whose training pathways may differ significantly across countries.

Using the regional health system database, a difference-in-difference case-control study is now being conducted between patients managed in CMI and those managed in the traditional care pathway to evaluate the efficacy and safety of the new organizational model in terms of subsequent hospital access and mortality over the next 30 days.

The number of patients evaluated in the CMI was low compared to the total number of patients referring to the EMS in the same time frame, probably due to the restrictive inclusion criteria and the unfamiliarity of first responders with this new alternative care pathway as well as the presence of a single physician per day. The extension of inclusion criteria and continuous education of the personnel of the EMS will probably increase the rate of non-urgent patients referred to the CMI, consequently requiring an increase in dedicated staff.

## 5. Conclusions

Using a telemedicine-based strategy to manage low-acuity patients referring to the EMS is feasible and potentially efficient and allows, in most cases, to avoid the evaluation in the ED. This strategy could improve the appropriateness of resource utilization in the pre-hospital and in-hospital Emergency Systems. A case-control study is needed to draw accurate conclusions about its efficacy and safety.

## Figures and Tables

**Table 1:** General exclusion criteria for CMI (investigated by first responders in SOREU).

GENERAL EXCLUSION CRITERIA
Age < 14 years Ongoing pregnancy ABCD alterations Active bleeding Suspected or confirmed intoxication Presence of a language barrier Event requiring intervention in an open space Intervention requested by medical personnel and law enforcement at the scene Unknown event dynamics

**Table 2:** Specific Inclusion and Exclusion Criteria investigated by first responders in SOREU.

SIGNS AND SYMPTOMS	INCLUSION CRITERIA	EXCLUSION CRITERIA
Confirmed or suspected COVID-19		Severe headache ≥10 episodes of vomiting/diarrhea in 24 h
Fever		Severe headache ≥10 episodes of vomiting/diarrhea in 24 h
Headache	Pain > 24 h	Fever Vomiting if first episode of headache
Non-traumatic limb pain	Present pain > 1 h	Cold skin on the limb
Spinal pain	Pain > 6 h	Trauma
Hypertension	180 mmHg < systolic blood pressure (SBP) < 150 mmHg 120 < diastolic blood pressure (DBP) < 95	Associated symptoms: chest pain/severe headache/dizziness/epistaxis
Vomiting/Diarrhea		≥10 episodes/day in the last 24 h
Blood glucose alterations	Blood glucose > 115 mg/dL	Blood glucose < 70 mg/dL
Trauma/Injuries to distal limb	Below the elbow/knee Minor dynamics (fall from own height, from chair, from bed)	Other body regions Lower back pain Caused by illness Active bleeding Major dynamics

**Table 3:** Main complaint of patients managed in the CMI.

Main Complaint	N° (%)
COVID-19	2542 (29.3)
Fever	2004 (23.1)
Other diagnosis	1731 (20)
Back pain	783 (9)
Vomit/Diarrhea	752 (8.7)
Pain in the limb	514 (5.9)
Hypertension	143 (1.6)
Headache	125 (1.4)
Glycemia abnormalities	86 (1)
Total	8680 (100)

**Table 4:** Main complaint of patients managed by the HRRT.

Main Complaint	N° (%)
COVID-19	537 (39.5)
Fever	338 (24.8)
Other diagnosis	178 (13.1)
Back pain	96 (7.1)
Vomit/Diarrhea	99 (7.3)
Pain in the limb	82 (6)
Hypertension	5 (0.4)
Headache	8 (0.6)
Glycemia abnormalities	8 (0.6)
Trauma	3 (0.2)
Vertigo	5 (0.4)
Total	1359 (100)

## Data Availability

The data presented in this study are available upon reasonable request from the corresponding author.

## Supplementary Materials

The following supporting information can be downloaded at: https://www.mdpi.com/article/10.3390/epidemiologia6030036/s1, Figure S1: COVID-19 Flow-chart embedded in CMI Software.

## Abbreviations

The following abbreviations are used in this manuscript:

EMS	Emergency Medical Systems
ED	Emergency Departments
LOS	length of stay
SOREU	Regional Emergency and Urgency Operation Centers
CMI	Integrated Medical Center
AREU	Regional Emergency and Urgency Operation Centers
HRRT	Home Rapid Response Team
POCUS	point-of-care ultrasound
PRU	Physician Response Unit
LAS EOC	London Ambulance Service Emergency Operations Centre
APP	Alternative Pre-Hospital Pathway
SpR	Specialist Registrar
NAS	National Ambulance Service
EMT	Emergency medical technician
