# Supplementary material for: Implementation of Telemedicine for Patients Referred to Emergency Medical Services
Source: Epidemiologia (Basel). 2025 Jul 11;6(3):36. doi: 10.3390/epidemiologia6030036 (PMC12286138; doi:10.3390/epidemiologia6030036)
Supplement: Supplementary file 1 [file epidemiologia-06-00036-s001.zip › epidemiologia-3733120-supplementary.pdf]

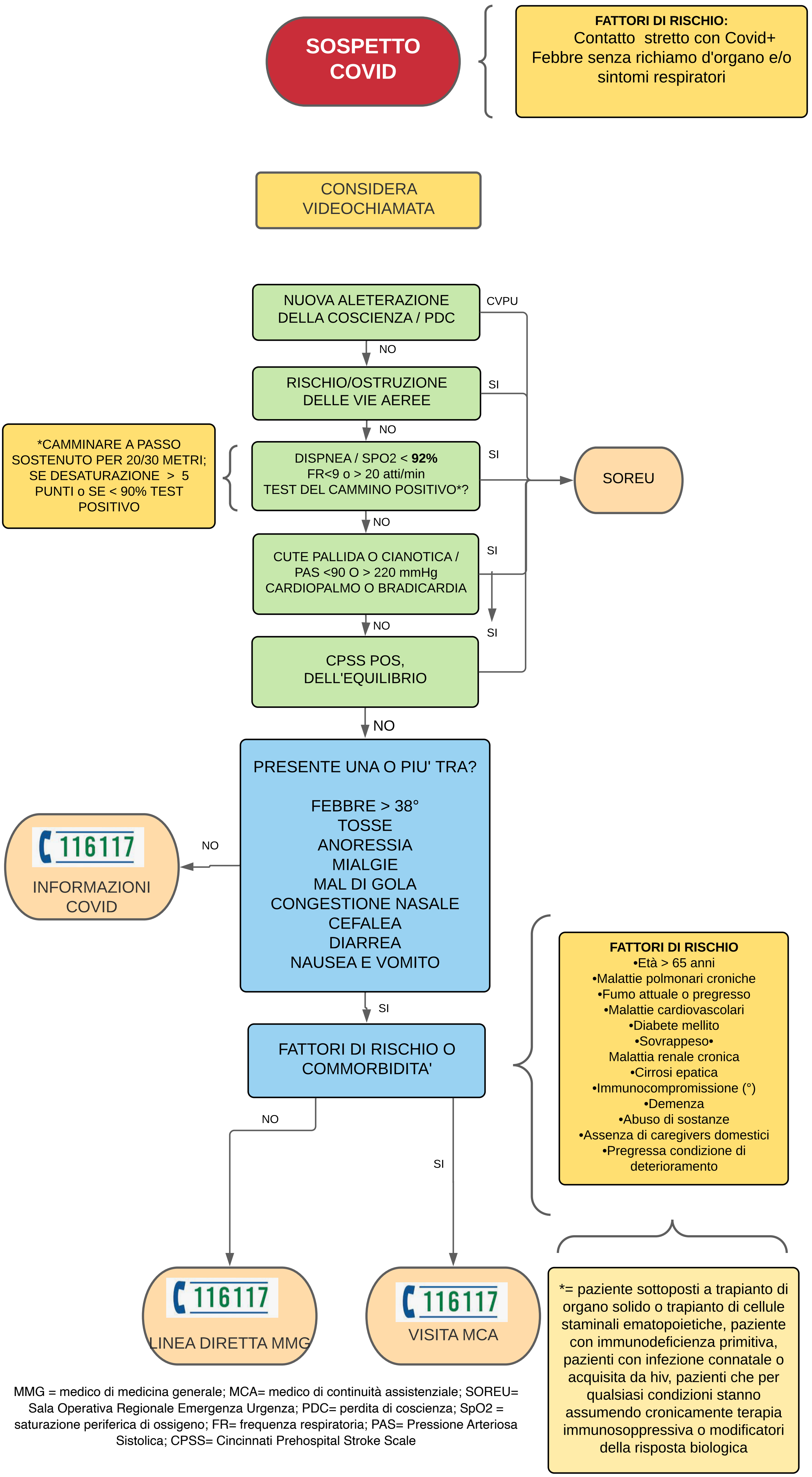

MMG = medico di medicina generale; MCA= medico di continuità assistenziale; SOREU= Sala Operativa Regionale Emergenza Urgenza; PDC= perdita di coscienza; SpO2 = saturazione periferica di ossigeno; FR= frequenza respiratoria; PAS= Pressione Arteriosa Sistolica; CPSS= Cincinnati Prehospital Stroke Scale
